# Medical Gossip

**Published:** 1861-10

**Authors:** 


### Art. XII.—MEDICAL GOSSIP.

A severe dignity surrounds the manner in which the Medical Council conducts its business, which must impress the profession with a very lofty sense of its wisdom. If it be the dignity of the pedagogue rather than that of the philosopher, this is, perhaps, the natural consequence of the peculiar functions delegated to the Council. It is pleasant, however, to find that it does not exempt itself from that stern adhesion to rule and precept which it is prepared to exact from others. We read, with a slight sense of awe, how in its last session it enacted that any member not present before the minutes of the previous day's meeting were confirmed, should be deemed absent; and that no member, after taking his place, should leave the meeting without permission of the chairman. Certain painful pedagoguish reminiscences immediately sprang up in our minds, and we felt assured—an assurance most fully borne out in the sequel—that the profession were once more about to be sent to school, under a rule that would admit of no relaxation, and which was based upon no pettifogging principles, but took its character from, and was formed after, the minds of its framers. There is nothing to regret, but much to rejoice at, in this prospect; yet there is some alloy. We are required to receive the collective wisdom of the Council in its most concentrated form—in its barest results only. In the true style of the ancient pedagogue, it is *Do this* and *Do that*, or *We think this*, *We think that*, but as to the *Why* or the *Wherefore* either of the direction or the opinion, we are left, as a rule, to the exercise of our own ingenuity. Now, with all deference to the majority of the members of the Council, we think that this is wrong, and that in the end, if persisted in, such a course must prove detrimental to the influence which the Council should at all times exercise over the minds of the profession. If the Council would work well, it must carry with it the majority of the profession. But how is this to be effected unless the latter are convinced that the decisions and suggestions of the former are both fit and just? To suffer the opinions of the profession on these important points to rest solely on the ingenuity of individuals, and to assume the privilege of a mere arbitrary dogmatism, is surely a dangerous policy. It is certain that by acting thus the Medical Council detracts from the influence which it ought to exercise over the profession, and that in the end its reasonings and conclusions must suffer from the want of that free criticism on the part of those whom these reasonings and criticisms most concern, and whom it is to be presumed have a chief interest in the matter. There may be many good reasons why reporters for the press should be excluded from the general meetings of the Medical Council, but there are many better why they should be admitted—at least, such we think is the soundest experience to be derived from the working of those public bodies which may be conceived to be analogous in their functions to those of the Medical Council.

Apart, however, from our tendency to grumble on this general question, the profession has much reason to be most highly gratified by the work effected by the Council in their last session. The recommendations it has made in reference to general and professional education go to the very root of the question of medical reform. It is only by raising the standard of the whole profession that the chief abuses which affect medicine in the present day can be eradicated, and it is through the student alone that this elevation can be permanently effected. The recommendation that all students should pass an examination in general education *before* they commence their professional studies, will, when carried into effect, be an immense step in advance. It will ensure the fitness of the student for the study of medicine, as far as this can be tested, and will for the future secure an average degree of mental training among students much higher than it has been customary to find or to require, and more befitting the acquisition of a complex profession.

The Council, while recommending this preliminary examination, do not lay down any complete scheme of general education for persons intending to become members of the profession, but they recommend that the scheme of examination in Arts of the licensing bodies should be, as nearly as practicable, similar to that of any one of the national educational bodies which they have specified (*e.g.* the Oxford, Cambridge, and Durham Middle-class examinations, &c.), testimonials of proficiency from which will exempt the holder from the preliminary examinations established by the licensing bodies. The Council, indeed, recommend that eventually the preliminary examinations should be left entirely to the examining boards of the national educational bodies which they name. No certificate of proficiency in general education will, however, be deemed sufficient, which does not affirm that the candidate has a competent knowledge of Latin; and after January, 1853, all junior Middle-class examinations will be excluded from the exemption list. The "time of commencing professional studies" is to be understood to be the "time of commencing studies *at a medical school.*" This important decision must prove a death-blow to the vile and degrading system of apprenticeship, by refusing to acknowledge it as a part of professional education.

The remaining recommendations of the Council on the subject of education, may be briefly summed up thus :—All students to be registered within fifteen days after the commencement of the winter session ; no professional licence to be granted at an earlier age than twenty-one years ; four years of professional study to be required after the examination in general education ; and the professional examinations to be divided into at least two distinct parts, the first not to be undergone until after two years' study, the second after the termination of the four years' study. It is suggested, moreover, that the examinations shall be conducted partly *vivâ voce*, partly in writing.

Most of the examining bodies have already anticipated to a greater or less extent the method of examination here recommended. Belonging to the time of cosy *vivâ voce* examinations, rarely exceeding an hour in duration, we compare with no small amusement the ordeal we ourselves were compelled to endure, with that which confronts the student of the present day, and which threatens the student of the future. How well we remember the pleasant quarter of an hour's chat with the honoured President of the Medical Council, at the College of Surgeons. How can we forget the kindness with which he proceeded to unfold the mysteries of a recondite physiological point, which in some manner or another had arisen out of his first question to us at the third table. Most grateful also is our remembrance of the genial tenderness with which Mr. Lawrence dealt with our little weaknesses, and of the agreeable gossip with Mr. Arnott. Mr. Arnott's examination took the form of an easy conversation of which, curious enough, one of the subjects was Mr. Teale, another member of the Medical Council. So chatty did the talk become that Mr. Arnott seemed to lose sight of the object of our being before him; and in answer to one or two questions from us he kindly gave us some very interesting information on one or two surgical points, when, as it were, suddenly bethinking himself, he exclaimed, infinitely to Mr. Guthrie's amusement, who sat along with him, and who in fact laughed outright, "It is you who are here for examination, sir, not I!"—or words to that effect.

Among the candidates who presented themselves for examination at the College of Surgeons the same evening as ourself, was a Licentiate of the Apothecaries' Company. We had met him once or twice before in the Secretary's room, while making the preliminary arrangements for our examination. On the evening of the examination, we had no sooner entered the College than this gentleman coming up to us, said, "By the way, do you know anything of the perinæum?" "Certainly," we answered. "Well," he rejoined, "I don't; I have not thought it worth while to get it up." "Not thought it worth while to get the perinæum up!" we exclaimed, with the most unfeigned amazement; "and offering yourself for examination." "Well," he said, "the fact is, the Examiners have been examining a good deal lately on the perinæum. Now they don't often question upon one subject long, lest students should get up specially for it; so I think the chances are against their asking much about the perinæum for a few nights, and consequently I've not bothered about it. Will you, then, if we're both out of the first batch, give me a little 'grind' on the subject?" "Certainly," I said, "I'll do what I can for you, but I suppose you have dissected the perinæum?" "Oh no," he coolly answered, "I dodged that." We were examined among the first batch of candidates; our extemporized friend was examined with the second. At the first table, fortunately, he was questioned on the perinæum, and of course broke down most wretchedly—in fact, he was not suffered to pursue the round of the Examiners. And so, for the time being at least, some unlucky locality was saved from the infliction of this ghastly specimen of unprincipled ignorance. We hope that the last days of persons taking their "chance" of passing in this fashion have gone by.

The final recommendation of the Medical Council on Education, "That it is not desirable that any University of the United Kingdom should confer any degree in medicine or surgery, whether that of Bachelor, Doctor, or Master, upon candidates who have not graduated in Arts, or passed all the examinations required for the Bachelorship in Arts, or the examinations equivalent to those required for a degree of Arts," has drawn a somewhat sharp response from the University of Edinburgh. The Senatus, while asserting that it would not yield to the Medical Council in anxious desire to improve and maintain the educational standard of medical graduates, whether in general or professional studies, suggests that different views might reasonably be taken of the best mode of attaining the object by different individuals. It expresses the opinion that "any such requirement in general studies as the Medical Council recommend, would be unequal, unfair, and even impracticable in many instances, so long as the same nominal degrees in Arts denote in different universities quite different standards of acquirement in the same branches, and even imply the study of different branches." It points out that the standard of examination for a degree in Arts of the Edinburgh University is now so high that it would be quite unreasonable to impose such an examination on candidates for a medical degree; and that were so high a qualification demanded of these candidates the result would be, not the advancement of the general education of medical graduates, but the necessity of lowering the standard for the degree in Arts. The Senatus then, "with great respect, takes the liberty" of reading the Medical Council a lesson. It wishes it to understand that, as becomes the dignity of an ancient University addressing a new-fangled Educational Board, it has noticed the recommendations of the latter simply as an act of courtesy. It conceives that the Council has gone beyond its jurisdiction in recommending to the Scottish Universities a different plan of education and examination from that which the University Commissioners have enjoined in their ordinances. The Medical Council is empowered by the Medical Act to take the necessary steps, according to a certain form of procedure, to secure, on the part of all persons presenting their qualification for medical practice to the registrars for admission into the register of legally-qualified practitioners, "the possession of the requisite knowledge and skill for the efficient practice of their profession." (Clause xx.) Now, the Senatus holds that the Medical Council have the right to demand this much of all graduates and licentiates of Universities and Medical Corporations alike and no more. "They are not empowered," says the Senatus, "to demand or recommend that, while the licentiates or corporations shall be qualified to that extent, graduates of universities shall be qualified in a higher degree; and the duty of fixing what that superiority should be, belongs to the authority of the Universities."

The Senatus is doubtless right in law and fact, but the Medical Council is certainly right in principle. The practical question is beset with many difficulties, but we do not fear that as the general standard of education in the profession advances, that of medical graduation, where below the level of our typical universities, must also advance.

*Apropos* of professional education, the institution of a degree in surgery (Master in Surgery), by the University of Cambridge, is a matter of considerable interest. Two of the English Universities, Durham and Cambridge, now give the degree of M.C. The examination for the surgical degree at Cambridge will be divided into two parts. The first will be the same as the first examination for M.B. degree, and the course of study for both degrees will be similar up to this point. The requirements for the second examination for M.C. will be anatomy, practical and surgical, pathology, and the principles and practice of surgery, clinical surgery, midwifery, and medical jurisprudence. The degree of M.C., as that of M.B., may be taken five years after admission to the University.

The chief gossip of the quarter is so intertwined with the proceedings of the Medical Council, that we cannot do better than take these still as our guide.

It is amusing to notice the little hitches that transpire from time to time, in the most perverse way, to the free working of the Medical Act. The Act provides that every person registered under it "shall be entitled, according to his qualification or qualifications, to practise medicine or surgery, or medicine and surgery, as the case may be, in any part of Her Majesty's dominions." It would appear, however, that a local Act exists in Canada, which requires all medical practitioners there to have the Governor-General's licence, and this is only granted to practitioners from the mother country if they possess a degree in medicine or surgery from a University, excepting only the licentiates of the London College of Surgeons, and that of the Dublin or Edinburgh College of Physicians—the former escaping the operation of the local Act by right of their privileges dating anterior to it, the latter by favour. Now, this provincial Act is proving very obnoxious to certain enterprising licentiates of the various licensing bodies who come within its restrictive scope, or, at least, one martyr has come to the surface, and the Medical Council has been called upon to interfere, and will no doubt do so effectually.

The *British Pharmacopœia*, it is most gratifying to learn, is approaching completion. The Pharmacopœia Committee of the Medical Council, in reporting upon the exact state of forwardness of the work, give us to understand that it will be divided into two parts, with an appendix. The first part is to contain a list of the *materia medica*, in which all the substances employed as medicines will be inserted, and appended to each will be its definition, and a statement of its origin, principal characters, the tests for purity, and an enumeration of its officinal preparations. The second part will contain the various groups of Galenicals, as extracts, infusions, tinctures, ointments, &c., with the method of preparing each, likewise the processes for making the numerous chemicals described in the first part of the work. The appendix is to include the substances employed, not as remedies, but only in their preparation, and likewise the various test solutions to be used in ascertaining the strength and purity of drugs. It is hoped that the *Pharmacopœia* will be published before the termination of the year; but the delay in its appearance, and seeming tardiness of compilation, is readily understood when it is known that every preparation in the work has been made, often repeatedly, and such process practically examined.

We may venture to hope that during the next session of Parliament some amendment will be made in the penal clause (XL.) of the Medical Act. As constituted, it is worse than useless. Several suggestions were made to the Medical Council, by which it was hoped that the difficulties that had arisen from the lax wording of the clause could be got over; but the Council will not proceed hastily in the matter. It is well that this should be so.

It will be a difficult task to weave the meshes of the legal net sufficiently close to catch the more dangerous charlatans, without at the same time narrowing them so greatly as to risk the danger of popular reaction on account of over straitness. For it is never to be forgotten that quackery is but an instance of popular credulity, and exists only on condition of the existence of that credulity. Hence measures for the restraint of quackery must, to be effective, proceed *pari passu* with measures calculated to diminish the credulity. But the means by which this is to be brought about are rather indirect than direct, and must depend greatly on the intellectual advancement of the people generally. The public weal is intimately concerned in the suppression of quackery, but unhappily the public has but a very restricted appreciation of the truth, and they will not learn it by any arbitrary measures. Morison and St. John Long lie beneath splendid mausoleums in Kensal-green cemetery, while one of the most distinguished orthodox physicians of the present century has but a simple mural tablet in the adjacent chapel. We must not lose sight of these facts, lest we should unwittingly convert disreputable quacks into *quasi*-reputable martyrs. The law must be made a means of disgraceful exposure as well as of suppression, if it is to be effective; for the suppression will in reality only be commensurate with the disgrace attending the exposure.

But, unfortunately, although illegal practice (whenever this may be verbally as well as morally defined) will include a vast mass of charlatanism, charlatanism is by no means confined to illegal practice. To see the fullest fledged charlatanism we need not step beyond the bounds of the profession. The most refined quacks stalk under cover of a legal qualification. The Register may, perhaps, be held *in terrorem* over the most arrant of these, but, after all, the only check upon them must be in the tone adopted by the body of the profession. We frankly confess to a species of respect for the bold charlatan who sets medical degrees and licences at defiance, and snaps his fingers openly at orthodox physic and physicians; but for the man who avails himself of a medical diploma or licence to cloak the practice of homœopathy, or hydropathy, or any other form of charlatanism, we have the most unmitigated contempt. In this case there can be no palliation; for if the man were honest he would put his diploma or licence aside, and the absence of honesty forbids usually the assumption that he acted from ignorance. The man is, in fact, a lie from beginning to end, and as such ought to be treated.

In the course of his examination in the Probate Court, Dr. Jones gave a curious history of his professional practice. He stated that although originally educated as an allopathist (by which we presume he meant as a practitioner according to the tenets usually held to be sound by our examining bodies), he had practised both homœopathy and hydropathy for a period of nine or ten years. Not, however, content with combining two systems of charlatanism, he had recourse to a third. He confessed to being the proprietor and patentee of a medicine called "*astramancax*," which he sold as a substitute for cod-liver oil. It was, moreover, elicited from him in the progress of cross-examination that his M.D degree from Marischal College, Aberdeen, had not been honestly acquired—that a friend of his, in short, had personated him at the College examination. This confession, as may be imagined, put a stop to the case, the witness's evidence being manifestly quite unworthy of credit.

This unvarnished story needs no comment, other than it shows the importance of the profession excising socially and, as far as possible, legally, all charlatans who prostitute the legitimate diplomas and licences of medicine.

The proposed amalgamation of several of the medical societies referred to in our "Gossip" of last quarter, has unfortunately fallen through, and the Medico-Chirurgical Society, its scheme having failed, has determined to effect an internal reform, by appointing Committees of Investigation from time to time.

Several questions of medical interest were brought before Parliament in the course of last session.

Lord Monteagle presented a petition from certain native Indian subjects of the Crown, complaining of their exclusion from the competitive examination for appointments to office; and he maintained the injustice and illegality of the exclusion. The question more especially arose out of, and referred to, the medical service of the Crown. Lord Herbert (whose loss the country has had so recently to regret), in reply, justified the exclusion both in law and reason. He stated that the Government had thrown open the right of competing for all appointments of a certain class in India to native-born subjects; but the claim of the petitioners was to be appointed surgeons and assistant-surgeons in line regiments liable to be stationed in all parts of the world. Now, the Directors-General of the Army and Navy Medical Departments, and Sir Ranald Martin, had, when called upon to give their opinion as to the fitness of Parsees for service in all quarters of the world, stated:—"It is our deliberate opinion, founded on experience, that the native and mixed race of India and other tropical countries will never be able to sustain, for any length of time, the climate of our Northern regions; and that they can only be employed advantageously to the public service in climates similar to those in which they exist." Lord Herbert thought this opinion conclusive, there being no legal claim of the petitioners to admission into the general service of the Queen.

A discussion was provoked in the House of Commons by a Bill introduced by Mr. Lowe to the effect that Poor-Law Boards might appoint persons to prosecute under the Vaccination Bill those whom the medical officers reported ought to be prosecuted, and to allow the expenses of prosecution to be paid out of the poor-rates, unless the magistrates should be of opinion that the information was improperly brought. The clauses proposed were somewhat modified in Committee, and the Bill passed.

The Lunacy Regulation Bill, introduced by Mr. Walpole, was arrested in its course, and the further discussion of the subject postponed.

The internal condition of the Irish District Lunatic Asylums was brought before the House of Commons by Mr. Blake. He drew an unfavourable comparison between the Irish and English public asylums, but he omitted to notice certain differences in the habits and social condition of many of the patients, which made the differences rather apparent than real, and that with a little ingenuity the comparison might be drawn in favour of the Irish asylums—a point not overlooked by the new Chief Secretary for Ireland, Sir R. Peel, in his reply. In the course of the discussion, which led to no results, Mr. Osborne paid a well-merited compliment to the "manly and able manner" in which Drs. Nugent and Hatchet had performed their duties as inspectors of lunatics.

The feud between the visiting physicians and managers of the Irish District Asylums has broken into open war. Both parties have appealed to the Lord-Lieutenant, but a fierce partisan warfare is being carried on in the meanwhile.

The Labourers' Cottages Bill, to which we have had occasion to refer in a previous number, was thrown out in the House of Lords.

Whatever difference of opinion there may be upon the question of special hospitals for the treatment of diseases, we presume that there will be none upon the propriety of founding a hospital for Incurables. These unfortunates have a great claim upon the consideration of all charitable people, and it is pleasant to record that the preliminary steps have been taken for founding an institution for at least a small number of them in the vicinity of London.

We may not do more than allude in the briefest way to the meeting of the National Association for the Promotion of Social Science at Dublin, under the presidency of Lord Brougham, and the meeting of the British Association, under the presidency of Mr. Fairbairn, at Manchester. Both meetings were great successes and at both medicine was most ably represented.

The British Medical Association also met at Canterbury, under the presidency of Dr. Lochée, of that town. The meeting was well attended, and appears to have off passed very pleasantly. The chief topics of discussion were consultation with homœopaths, and special hospitals. The Association passed a series of resolutions condemnatory of the former, and calculated to purge the Association of any heretical members, and gave up the latter in despair.

A sadder subject furnishes the last note of our gossip. It is hardly too much to say that but one pleasant feature has become apparent in the fratricidal war which now convulses the United States. The energy with which our American medical brethren attached to the forces in the field have met the enormous sanitary evils arising out of the rapid formation and necessarily incomplete camp equipment of volunteer corps, and the admirable manner in which they have kept at their posts, even in the horrid turmoil of defeat, is beyond all praise. After the disastrous action at Manassas, several of the Federal surgeons remained on the field, attending to the wounded, after the retreat of their regiments, and of course fell into the hands of the Confederate troops.

The medical men have been nobly aided in the hospitals by the American ladies, and a Miss Dorothea Dix, who has been termed by one of her contemporaries "a monomaniac in philanthropy"—one of those unfortunate feminine sneers which become a golden testimony to single-minded nobility of aim—is desirous of emulating the deeds of Miss Nightingale.

---

### Art. XIII.—LITERARY GOSSIP AND RECORD.

